# Urinary detection of corticosteroid in topical treatment of skin disease by ^19^F MRS

**DOI:** 10.1007/s10334-018-00734-y

**Published:** 2019-01-04

**Authors:** Beathe Sitter

**Affiliations:** Department of Circulation and Medical Imaging, Faculty of Medicine and Health Sciences, NTNU – Norwegian University of Science and Technology, Trondheim, Norway

**Keywords:** Renal, Excretion, Treatment, Skin, Betamethasone 17-valerate

## Abstract

**Objective:**

To investigate if it was feasible to quantify the renal excretion of topically applied corticosteroids by ^19^F MRS.

**Materials and methods:**

Five participants, one healthy and four with skin diseases, were treated with ointment containing betamethasone 17-valerate. Urine samples were collected for up to 87 h after the initial application. A sample of ointment mixed with urine served as a study control. Organic fractions were obtained after sample freeze drying, and resolved in deuterated chloroform prior to acquisition of ^19^F MR spectra at 470 MHz for typically 8 h.

**Results:**

We detected fluorine signals in 40 of the 62 fractions of organic extracts. The corticosteroid was detected in samples from all patients, ranging from 0.1 to 2.8% of the applied steroid. No fluorine signal was obtained in samples from the healthy volunteer.

**Discussion:**

^19^F MRS can be utilized to detect topically applied corticosteroids in urine. However, more work is required to optimize and control for extraction procedures, complete spectral assignments and reliable quantification.

## Introduction

Topical application of corticosteroids is the first and main treatment for many skin diseases, like psoriasis, and has been used since the mid-60s [1, 2]. Their main actions are anti-inflammatory, antiproliferative, immunosuppressive and vasoconstrictive, mediated through binding to intracellular receptors and regulation of gene transcriptions [3]. The molecular mechanisms of improvements in skin conditions due to corticosteroid treatment are still partly unknown [4]. Treatment is periodic, as long-term corticosteroid application leads to skin thinning and inhibition of the cortisol production. Both the effectiveness of different treatments in psoriasis and comparative effectiveness for clinical variants should be studied more [3].

The skin absorption and bioavailability of corticosteroids are low, typically only a few percentages of applied dose [5, 6]. Several factors determine this, importantly the chemical structure of steroid, the concentration of applied corticosteroid, choice of vehicle, and thickness and health condition of skin [3]. After absorption in skin, topically applied corticosteroids undergo pharmacokinetic pathways similar to systemically administered corticosteroids [7]. They are primarily metabolized in the liver and excreted by the kidneys. Some corticosteroids and their metabolites are also excreted in the bile. The quadruple ring structure is not degraded in this process of metabolization. The most common modifications are additions of hydroxyl groups, reduction of carbonyl groups and ring methylations, and the corticosteroid and its metabolites can be found in urine as conjugates and free steroids.

**Fig. 1:**
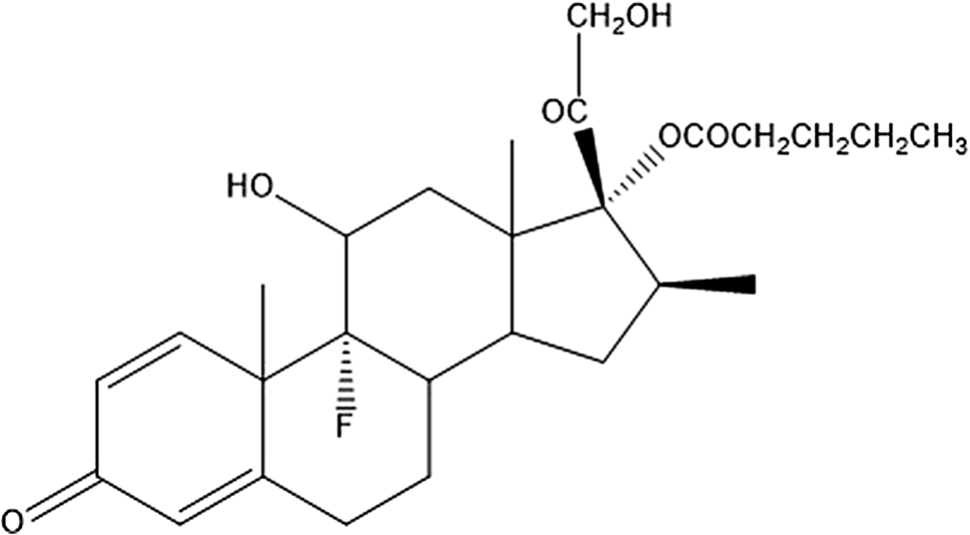
Chemical structure of betamethasone 17-valerate (BMV), with a fluorine atom in the 9α position

Corticosteroid with a fluorine atom is the active compound in several common ointments for topical treatment of skin diseases, as preparations with the highly potent betamethasone 17-valerate (BMV) with one fluorine atom in 9α position (BMV, Fig. 1). Fluorine is a body trace element, and signals in urine by ^19^F MRS after skin treatment would, thus, origin from the topically applied corticosteroid. ^19^F MRS of urine samples from patients after topical treatment with fluorinated corticosteroids can provide a novel method to gain knowledge on corticosteroid metabolism. This can be related to treatment response and side effects, and adds to our knowledge of corticosteroid mechanisms of action. However, identification and quantification in urine may be challenging due to low absorption and extensive metabolization.

The purpose of this pilot study was to investigate if it was feasible to quantify the renal excretion of topically applied corticosteroids by ^19^F MRS. Five study participants with healthy skin (*N* = 1) and with skin conditions (*N* = 4) were subject to the same topical treatment with BMV. Urine samples were collected for up to 4 days after start of treatment, and organic extract fractions were analyzed by ^19^F MRS. A study control was performed by submitting a mixture of topical ointment in urine to the same laboratory procedures as urine samples from patients.

## Materials and methods

### Study participants

Study participants (*N* = 5) were recruited at St. Olav’s University Hospital, Department of Dermatology, Trondheim, Norway. One participant (male, 55 years) was a volunteer without any skin disease. Four participants (P1–P4) had a skin disease [psoriasis (*N* = 2), peuritas (*N* = 1) or contact dermatitis (*N* = 1)], and were in hospital for topical corticosteroid treatment. None were under treatment the last 2 weeks prior to the study. All participants were topically treated with ointment (Betnovate, Glaxo) with BMV (0.122% w/w) according to guidelines [3]. Patients received topical treatment once (*N* = 1) or twice (*N* = 3) per day. The healthy volunteer received only one treatment. We weighed the tube before and after applications to calculate applied amount of corticosteroid. Data on study participants and topical applications of BMV are given in Table 1.

**Table 1:** Characteristics of study participants (healthy volunteer and patients 1–4), and the topical treatment with betamethasone 17-valerate (BMV)

	Healthy volunteer	P1	P2	P3	P4
Gender	M	M	M	F	F
Age	55	44	55	68	68
Skin condition	None	Psoriasis	Psoriasis	Peuritas	Contact dermatitis
Number of BMV applications	1	5	7	2	7
Time-span for applications of BMV (h)	< 1	47	74.33	24	74
Total amount of applied BMV (10^−6^ mol)	39.90	88.20	279.30	35.70	249.90

Informed consent was obtained from all individual participants included in the study.

### Sample preparations

Urine samples were collected from start of treatment over different time-spans (26.75–87.25 h). Table 2 displays detailed data on the urine samplings. In total, 62 samples were included in this study. In addition, ointment (6.63 g, Betnovate, Glaxo) with BMV (0.122% w/w) mixed with urine from healthy volunteer (80 ml) served as a study control sample. Samples were stored at − 20 °C. Each sample was further cooled down prior to vacuum chamber freeze-drying by adding liquid nitrogen (N_2_ (l), − 196.7 °C). The freeze-dried fractions were added sodium chloride (NaCl, 15 g), solved in distilled water (15 ml) and transferred to separating funnel through filter paper. Each fraction was extracted 10 times with dichloromethane (CH_2_Cl_2_, 15 ml). The resulting organic fractions were dried over magnesium sulfate (MgSO_4_·H_2_O), and transferred via filter to evaporator flasks before solvent removal over 40 °C water bath in a rotary evaporator. Each of the organic fractions was transferred to 5-mm NMR tubes (Bruker Biospin, Germany) by deuterated dichloromethane (CDCl_3_, 0.5 ml) and added hexafluorobenzene (C_6_F_6_, 4.35 nmol) as reference.

**Table 2:** Timespan, number and volumes of sampled urine from the five participants

	Healthy volunteer	P1	P2	P3	P4
Hours of urine sampling	26.75	51.33	87.25	44.17	79.25
Number of urine samplings	1	11	32	6	12
Total volume of urine (ml)	935	3562	8298	1732	6555

Separating funnel and filters for each sample were rinsed with distilled water (10 ml), which were added to one total water fraction for each participant. Water fractions were freeze-dried after freezing in liquid nitrogen, and finally dissolved in heavy water (D_2_O, 4 ml). 0.5 ml was transferred to 5-mm NMR tubes (Bruker Biospin, Germany).

## ^19^F MRS acquisitions

All urine extracts were analyzed on a Bruker AM500 with a 5 mm ^1^H/^19^F dual probe. Spectra were acquired using a 45° flip angle, 1.64-s repetition time, ^1^H decoupling and sample temperature of 23.8 °C. Number of acquisitions were in average 19,270 (8400–32,500), and spectra were typically recorded over 8 h. Spectrum of ointment–urine mixture was acquired using 4 K scans. Raw data were Fourier transformed to 128 K points after 10 Hz exponential line-broadening. The spectral peaks were integrated after spectral baseline adjustment. All fluorine signals were interpreted as BMV or its metabolites. Quantification of BMV and its metabolites was based on integrals relative to hexafluorobenzene. We calculated the total amount of detected BMV and metabolites for each sample and for each patient.

## Results

**Fig. 2:**
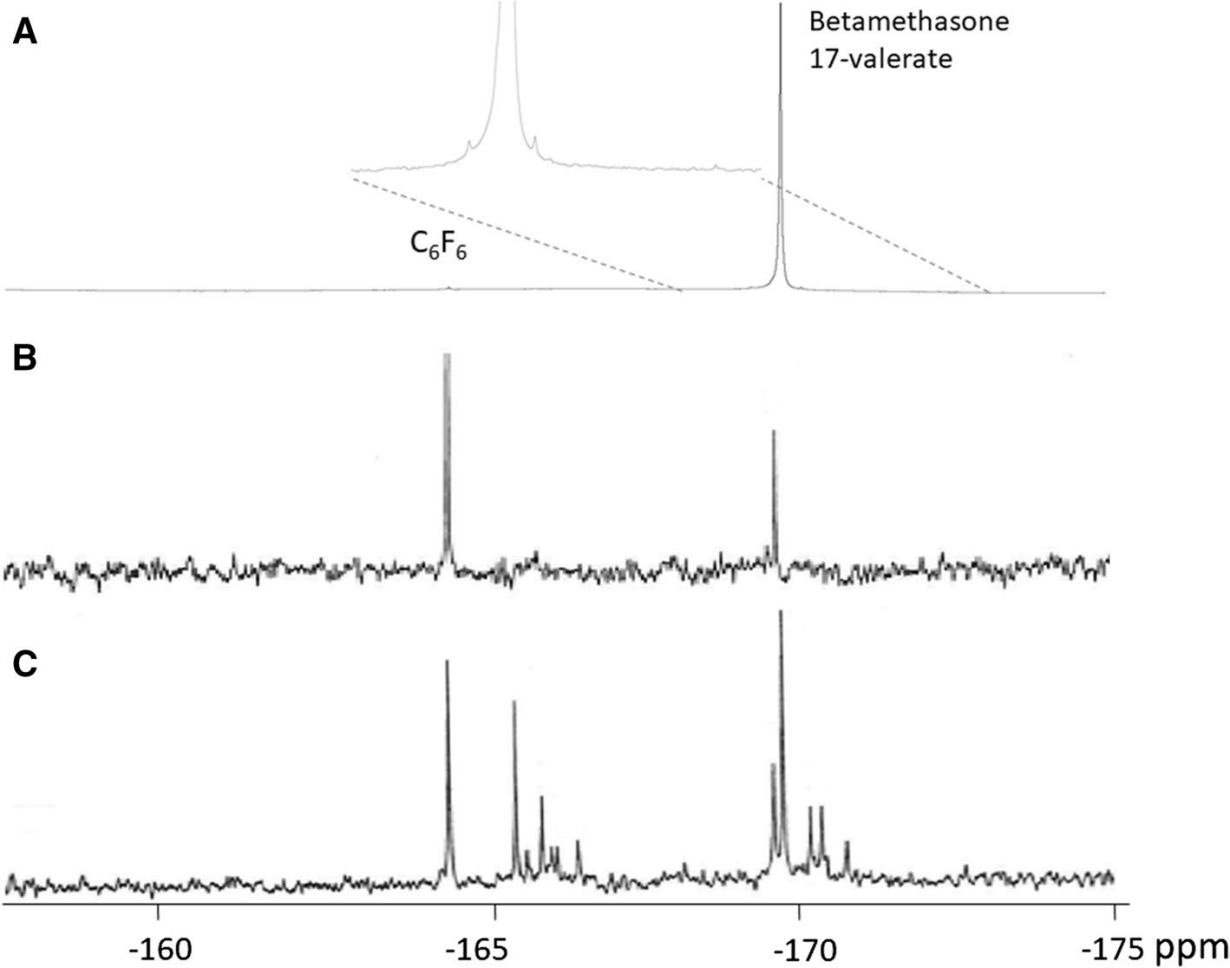
^19^F MR spectra of organic extract fraction from (A) betamethasone ointment mixed with urine, with expanded region − 168 to − 173 ppm, (B) patient number 4 at 79.2 h and (C) patient number 2 at 15, 5 h after first application of corticosteroid. Chemical shift is referenced to hexafluorobenzene (C_6_F_6_) at − 164.9 ppm. The fluorine in 9α of betamethasone 17-valerate was assigned to the resonance at − 169.9 ppm

We detected fluorine signals in 40 of the 62 ^19^F MR spectra of organic extract fractions. There was no detectable fluorine signal in water fractions (results not shown). The detected fluorine resonances were in the chemical shift region − 165.5 and − 172.5 ppm, with BMV assigned to the resonance signal at − 169.9 ppm. Up to 12 different ^19^F resonances were detected in one organic extract fraction (Fig. 2). The study control spectrum is dominated by one peak at − 169.9 ppm, and additional three small peaks (< 1% of main peak) can be observed.

**Fig. 3:**
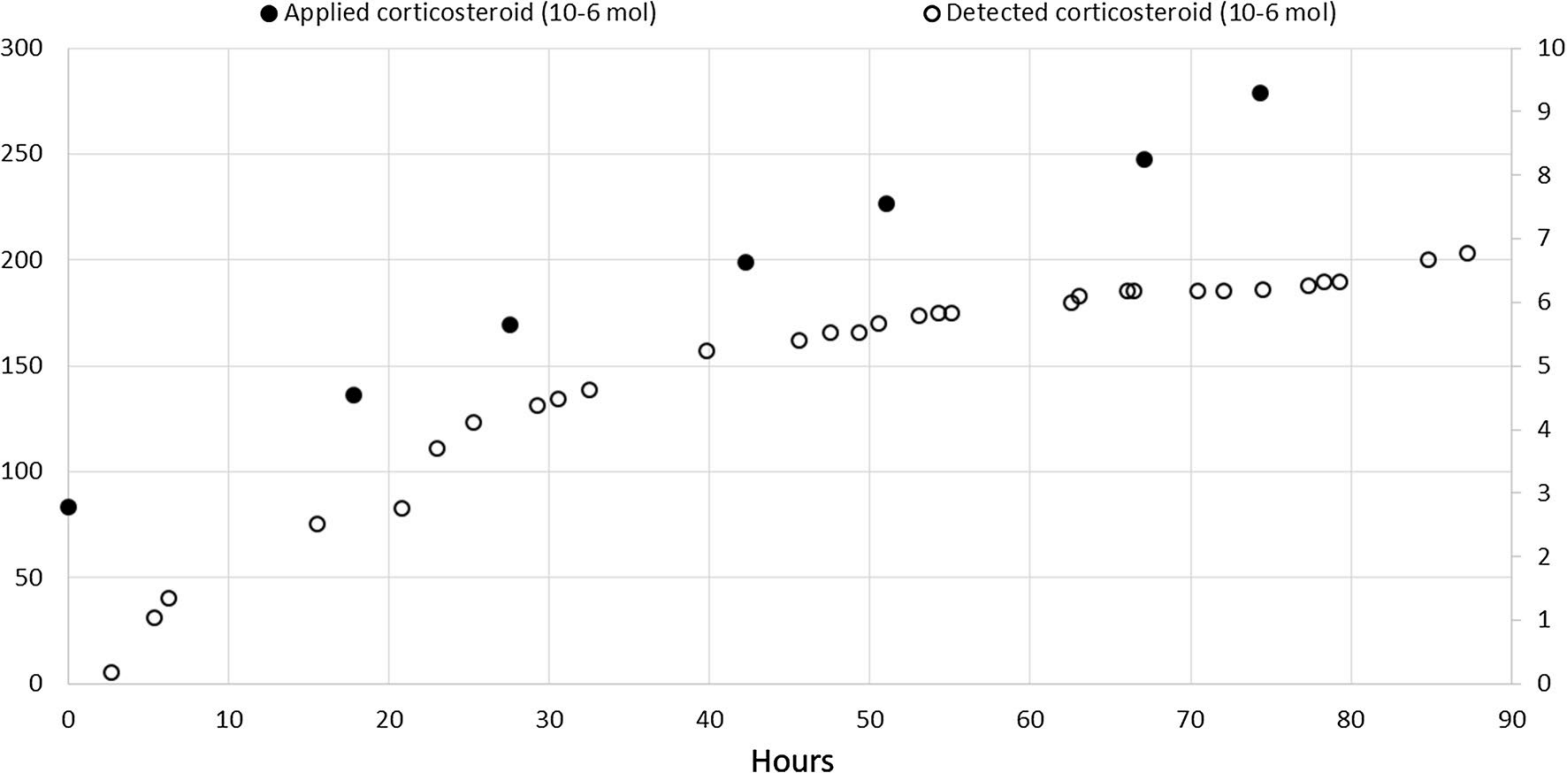
Applied (filled circles, left *y*-axis) and detected (open circles, right *y*-axis) added amounts of betamethasone 17-valerate (BMV, μmol) in patient number 2 as a function of time. BMV was applied seven times over 74.3 h, whereas urine was collected 32 times over 87.25 h

BMV or its metabolites were found in urine samples from all patients, but not in the urine from the healthy volunteer. The total detected portion of topically applied BMV varied from 0.1 to 2.8% in total urine from the four patients (Table 3). The proportion of total applied to total detected BMV remained fairly constant over time. This is illustrated in Fig. 3, where total applied and total detected BMV is plotted as a function of time for patient number 2.

**Table 3:** Detected total amounts of betamethasone 17-valerate (BMV) and its metabolites, and fraction of applied BMV, in urine samples from the five participants

	Healthy volunteer	P1	P2	P3	P4
Hours of urine sampling	26.75	51.33	87.25	44.17	79.25
Detected BMV and metabolites (10^−6^ mol)	0.00	0.12	8.21	0.28	1.28
Detected fraction of applied BMV (%)	0.0	0.1	2.9	0.8	0.5

## Discussion

We detected BMV and up to 11 different metabolites in urine samples after topically applied BMV (Fig. 2). The ^19^F MR spectrum of the study control sample demonstrates that modifications may occur due to the experimental procedures, but that more than 99% of the steroid apparently remains as unaltered BMV. Figure 2C shows the ^19^F MR spectrum with most fluorine resonances, which was a sample from patient number 2 taken 15.5 h after the first application of BMV. There is a group of fluorine resonances close to the signal from BMV, mainly shifted down-field. These compounds have chemical modifications with minor effects on the chemical shift of the 9α fluorine atom. The group of signals at − 166 to − 167 ppm are shifted up-field from BMV. It is plausible that BMV undergoes the same modifications as betamethasone [8]. However, the information on the ^19^F chemical shift alone is insufficient to depict structures of the BMV metabolites. To identify the different compounds in urine samples, spiking with reference compounds, ^19^F MRS without ^1^H decoupling and two-dimensional hetero-nuclear spectroscopic methods would be required.

Very small and variable fractions (0.1–2.8%) of the applied BMV were detected in the urine from patients (Table 3). The values were in accordance with previously reported skin permeability for topically applied corticosteroids [5, 6, 9]. The observed lower absorption in healthy skin is also in accordance. It has been reported that < 2% of topically applied hydrocortisone was absorbed into systemic circulation [6]. Also, Kao and Hall investigated the permeability for five selected steroids in mouse skin, where the permeation varied from 65.1% for testosterone, to 2.45% for estriol [9]. Absorption of BMV in human skin, thus, seems to be similar to that of estriol on mouse skin.

Possible sources of inaccuracies in quantification of BMV and its metabolites are (1) slightly lower amounts of applied corticosteroid than calculated based on weights of sample tubes, (2) loss of material in the freeze-drying and extraction procedures, and (3) errors in spectral peak integral values and external referencing by hexafluorobenzene. The extraction of corticosteroid and derivatives to organic fractions is complete, supported by no detected fluorine in the water fractions. Some loss of material in the experimental procedures is likely. Furthermore, low signal-to-noise in the ^19^F MRS spectra led to inaccuracies in peak integral values, and the volatile nature of hexafluorobenzene brings a risk of error in calculations of concentrations. It is, thus, likely that the observed variations between patients partly are due to inaccuracies in calculations of amounts of BMV and its metabolites. But, as the reported amounts of BMV in urine in this study are compatible with previously reported values [5, 6, 9], the observed variations can also partly be explained by the differences between study participants.

The largest difference in detected fraction of applied BMV (0.1% vs 2.8%) was observed between patient number 1 and 2 (Table 3). Both were men diagnosed with psoriasis, with urine samples collected for 51 and 87 h, respectively. The two female patients (number 3 and 4) were older (68 years), with different diagnosis, and with intermediate detected fractions of applied BMV. The available data are inadequate to explain the differences observed between study participants. But, these results support that factors other than gender and skin condition might have big impact on the pharmacokinetics of topically applied corticosteroid [10].

As the concentrations of corticosteroid and its metabolized forms in urine are low, the protocol should be optimized for sensitivity. Urine sampling could be timed to the highest levels of excreted corticosteroids after application time-points, and samples from multiple time-points should be added. Extraction of dichloromethane was time-consuming, and extraction protocols could be tested to ensure a reliable but faster procedure [11]. The protocol should maintain the chemical structure of the steroid. Furthermore, T_1_ measurements of the fluorine nucleus in the corticosteroid will allow for optimized acquisition time. Higher field strengths and cry-probe are recommended to further increase sensitivity.

The major advantage of ^19^F MRS is that it is highly specific for the applied corticosteroid. NMR spectroscopy can, thus, provide insight to the metabolism of topically applied corticosteroids. As a tool in studies of effectiveness of various corticosteroids in one skin condition, or in comparisons of effectiveness for clinical variants of skin disease, 19F MRS of urine could generate new knowledge on both treatment and side effects from topically applied glucocorticoids. This pilot study demonstrates the potential of ^19^F MRS in this respect, but more work is required to provide quantitative and reliable measures of the pharmacokinetics of topically applied corticosteroids.

## Conclusion

This pilot study demonstrated that topically applied BMV in patients with skin disease could be quantified in urine by ^19^F MRS. Less than 3% of the topically applied corticosteroid was detected. Eleven different metabolites of the corticosteroid were detected in the urine samples.
